# Structural insights into Cdc45 function: was there a nuclease at the heart of the ancestral replisome?

**DOI:** 10.1016/j.bpc.2016.11.011

**Published:** 2017-06

**Authors:** Luca Pellegrini

**Affiliations:** Department of Biochemistry, University of Cambridge, Cambridge CB2 1GA, UK

## Abstract

The role of Cdc45 in genomic duplication has remained unclear since its initial identification as an essential replication factor. Recent structural studies of Cdc45 and the evolutionarily-related archaeal GAN and bacterial RecJ nucleases have provided fresh insight into its function as co-activator of the MCM helicase. The CMG helicase of the last archaeal/eukaryotic ancestor might have harboured a single-stranded DNA nuclease activity, conserved in some modern archaea.

## Highlights

• The structure of human Cdc45 provides insight into its function in DNA replication as co-activator of the MCM helicase.
• Structural analysis of human Cdc45 and bacterial RecJ illustrates the structural elements of their shared nuclease fold.
• The structure of the archaeal GAN nuclease shows that it is an orthologue of eukaryotic Cdc45.
• The Cdc45 subunit of the ancestral predecessor of archaeal and eukaryotic CMG helicases was a single-stranded DNA nuclease.

## Graphical abstract

**Figure:**
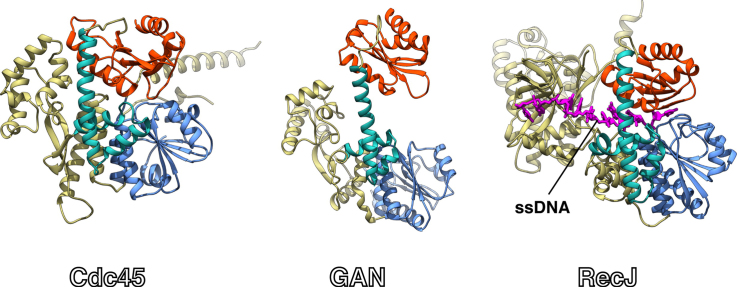

## 1 Introduction

Activation of the MCM helicase on DNA replication origins at the beginning of S-phase requires the recruitment of two accessory factors, the GINS heterotetramer and Cdc45, in a carefully controlled process of assembly that results in formation of the Cdc45-MCM-GINS (CMG) helicase complex [1], [2], [3]. The incorporation of GINS and Cdc45 enhances the biochemical ability of the MCM AAA ATPase to unwind DNA and therefore to act as an efficient helicase motor during DNA replication [4].

Molecular insights into Cdc45 function had remained limited until bioinformatics analysis demonstrated a similarity of its N-terminal amino acid sequence with the DHH domain of bacterial RecJ, a 5′-to-3′ single-stranded (ss) DNA exonuclease that functions in recombinational DNA repair [5], [6], [7]. Low-resolution negative-stain electron microscopy (EM) studies of the CMG had shown that GINS and Cdc45 bind to the same side of the MCM ring, acting jointly to form a brace across the dynamic interface between MCM subunits 2 and 5 [8]. Together, these observations were combined in mechanistic models which proposed that Cdc45’s DNA-binding ability might be harnessed to entrap the escaping leading DNA strand during CMG helicase stalling or partial disassembly [9], [10].

Several recent high-resolution studies have now advanced considerably our structural understanding of eukaryotic Cdc45 [11], as component of the CMG helicase [12], [13], and of its related structural orthologues, the bacterial RecJ nuclease [14] and the archaeal GINS-associated nuclease (GAN) [15], also known as RecJ-DNA-binding domain homologue (RecJdbh) [16]. Here I will review these advances and summarise how they help us understand the function of Cdc45 in eukaryotic DNA replication.

## 2 Structure of human Cdc45 and implications for CMG function

**Fig. 1:**
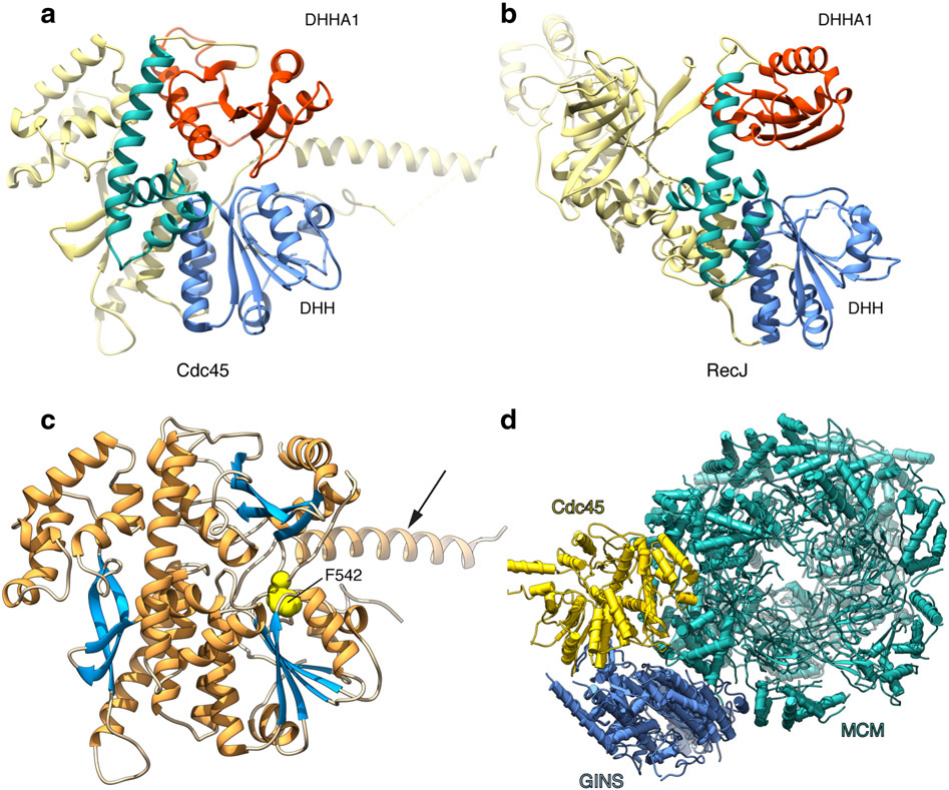
High-resolution structures of human Cdc45, bacterial RecJ and yeast CMG. a Crystal structure of human Cdc45 (PDB ID 5DGO). The structure is drawn as a ribbon diagram, and coloured to highlight its Cdc45/RecJ fold: the N-terminal DHH domain is in light blue, the C-terminal DHHA1 domain in red and the 3-helix domain connecting the two DHH domains in green. b Crystal structure of *Thermus thermophilus* RecJ (PDB ID 2ZXO). The structure is drawn as ribbon and coloured as for Cdc45. c Invariant amino acid F542 of human Cdc45 tethers the DHH and DHHA1 domains together. The structure is drawn as a ribbon and coloured according to secondary structure (alpha helices in orange and beta strands in light blue). The F542 side chains is coloured yellow, in spacefill representation. The arrow points to the helical insertion protruding from the DHH domain. d Cryo-EM structure of yeast CMG (EMDB ID 6535; PDB ID 3JC5). The structure is shown as a ribbon diagram, and the alpha helices as cylinders. The MCM ring is coloured in green, GINS in light blue and Cdc45 in yellow.

High-resolution crystallographic analysis of human Cdc45 has confirmed the bioinformatics prediction of similarity with the RecJ N-terminus [11] (Fig. 1a). Comparison of the atomic models for human Cdc45 and bacterial RecJ has elucidated the core elements of their evolutionary conserved fold, comprised of an N-terminal DHH domain, a C-terminal DHHA1 domain and a connecting three-helix bundle [11] (Fig. 1b). The C-terminal helix of the bundle is considerably longer than the other two and acts as a flexible stem or strut between DHH and DHHA1 domains.

The active site of RecJ is located in the N-terminal DHH domain, within a groove formed by the juxtaposition of the two DHH domains. In the case of Cdc45, the DHH residues coordinating the catalytic metal ions are found to be consistently mutated, and therefore Cdc45 has no catalytic activity. An unexpected feature of the structure of human Cdc45 is the presence of an intra-molecular tether between DHH and DHHA1 domains, formed by the insertion of invariant DHHA1 residue F542 into a hydrophobic pocket on the DHH surface (Fig. 1c). As a result of this interaction, access to the inter-domain groove, where Cdc45's active site is located, is effectively blocked. It is not clear at present what could be the functional role of this interaction. It is possible that tethering DHH and DHHA1 in this fashion confers increased stability to Cdc45's tertiary structure, which might be a requirement of replisome architecture. Alternatively, access to the inter-domain groove of Cdc45 might be regulated and only possible under specific circumstances.

A second striking aspect of the Cdc45 structure is the unusual conformation of a large insertion present in the C-terminal of the DHH domain (Fig. 1c). The insertion consists of a single, long helix projecting from the globular core of Cdc45, and preceded by a largely disordered acidic sequence of amino acids. EM studies of the CMG show that this helical insertion points away from the bulk of the CMG [8], [13]; its functional role is not known, but it might represent a buttress for further interactions within larger CMG assemblies, or possibly act as a lever, to mediate conformational changes in replisome architecture.

Recent cryoEM analyses of yeast and fly CMG have elucidated the relative position of Cdc45 within the helicase assembly and, together with crystallographic data, helped define its interaction interfaces with MCM and GINS [12], [13] (Fig. 1d). Cdc45 binds to the N-terminal tier of the MCM ring, contacting MCM subunits 2 and 5. The interaction is mediated by a region of Cdc45 that lies outside the conserved RecJ/Cdc45 fold, termed CMG-interacting domain or CID, located after the N-terminal DHH domain and before the three-helix bundle of the RecJ/Cdc45 fold. Conversely, the interaction with GINS affects a distinct site on the Cdc45 surface, mainly comprising its N-terminal DHH domain. Neither of the two interfaces appears sufficiently large to confer stability to a binary Cdc45-MCM or Cdc45-GINS interactions, in support of the current step-wise model of CMG assembly requiring the regulated intervention of conserved replication factors such as Sld3/Treslin to chaperone Cdc45 to the helicase ring.

## 3 The GAN nuclease as the archaeal Cdc45

**Fig. 2:**
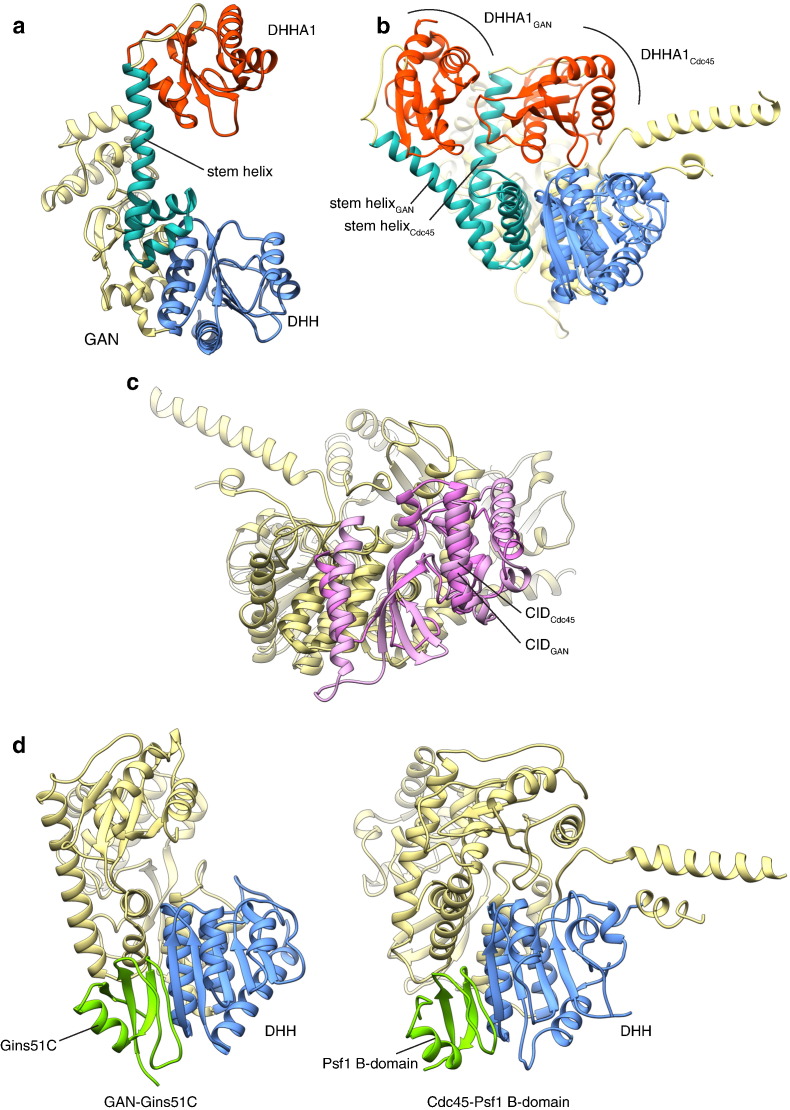
Comparison of human Cdc45 and archaeal GAN. a Crystal structure of *Thermococcus kodakaraensis* GAN (PDB ID 5GHT), shown as a ribbon diagram, and coloured to highlight its similar tertiary structure to Cdc45 and RecJ, as in Fig. 1. b Superposition of human Cdc45 (PDB ID 5DGO) and archaeal GAN. The flexible helical stem connecting DHH and DHHA1 domain has a different conformation in Cdc45 and GAN, leading to different positions of their DHHA1 domains relative to the rest of their structure. c Superposition of human Cdc45 and archaeal GAN, highlighting the similarity of their CMG-interaction-domains (CIDs; drawn in two hues of pink). d Side-by-side comparison of the crystal structure of GAN bound to the C-terminal domain of Gins51 (left; PDB ID 5GHS) and yeast Cdc45 bound to the Psf1 B-domain, as observed in the structure of yeast CMG (right; (EMDB ID 6535; PDB ID 3JC5). The structures are shown in ribbon diagram, with the DHH domains in light blue and the Gins51C and PSf1 B-domain in light green.

An important piece of the Cdc45 puzzle was solved recently with the determination of the crystal structure of the archaeal GAN, alone and in complex with the C-terminal domain of the Gins51 subunit (Gins51C) [15] (Fig. 2a). GAN was originally reported as a processive 5′-to-3′ ssDNA exonuclease in *Thermococcus kodakaraensis*, which bound to and was stimulated by the Gins51 subunit of the GINS heterodimer [17]. Interestingly, structural comparison of human Cdc45 and archaeal GAN highlights the flexibility of the alpha helical stem that connects the DHH and DHHA1 domains; in the GAN structure, the helix is bent backwards to a considerable degree, likely as result of crystal packing, leaving GAN's DHH active site fully exposed (Fig. 2b). Overall, the crystal structure of GAN shows a remarkable structural similarity with that of human Cdc45, including the presence of a complete CMG-interaction domain (Fig. 2c). Furthermore, the structure of GAN bound to the Gins51C reveals that their mode of interaction is essentially identical to that observed between Cdc45 and the C-terminal domain of GINS subunit Psf1 in the eukaryotic CMG [13] (Fig. 2d).

These biochemical and structural observations suggest that the CMG helicase assembly of *Thermococcus kodakaraensis*, as well as the CMG of other archaeal organisms possessing GAN orthologues [18], harbours an active ssDNA exonuclease as one of its constitutive components. Some important questions arise from this surprising finding: what is the functional role of the nuclease component of the archaeal helicase? As Cdc45 is inactive, what eukaryotic nuclease might have superseded a putative ancestral nuclease activity of Cdc45? Locating a ssDNA nuclease near the site where dsDNA is unwound is potentially hazardous, so presumably GAN activity must be tightly regulated, either by keeping GAN in an inactive conformation, or by controlling the path of the DNA strands as they emerge from the helicase, to avoid accidental cleavage. Conversely, any functional requirement for cleavage or resection of the DNA template during DNA replication would conceivably require a specific conformational rearrangement of the archaeal replisome, re-directing the trajectory of the DNA towards the nuclease.

## 4 The RecJ - ssDNA structure and implications for GAN and Cdc45

**Fig. 3:**
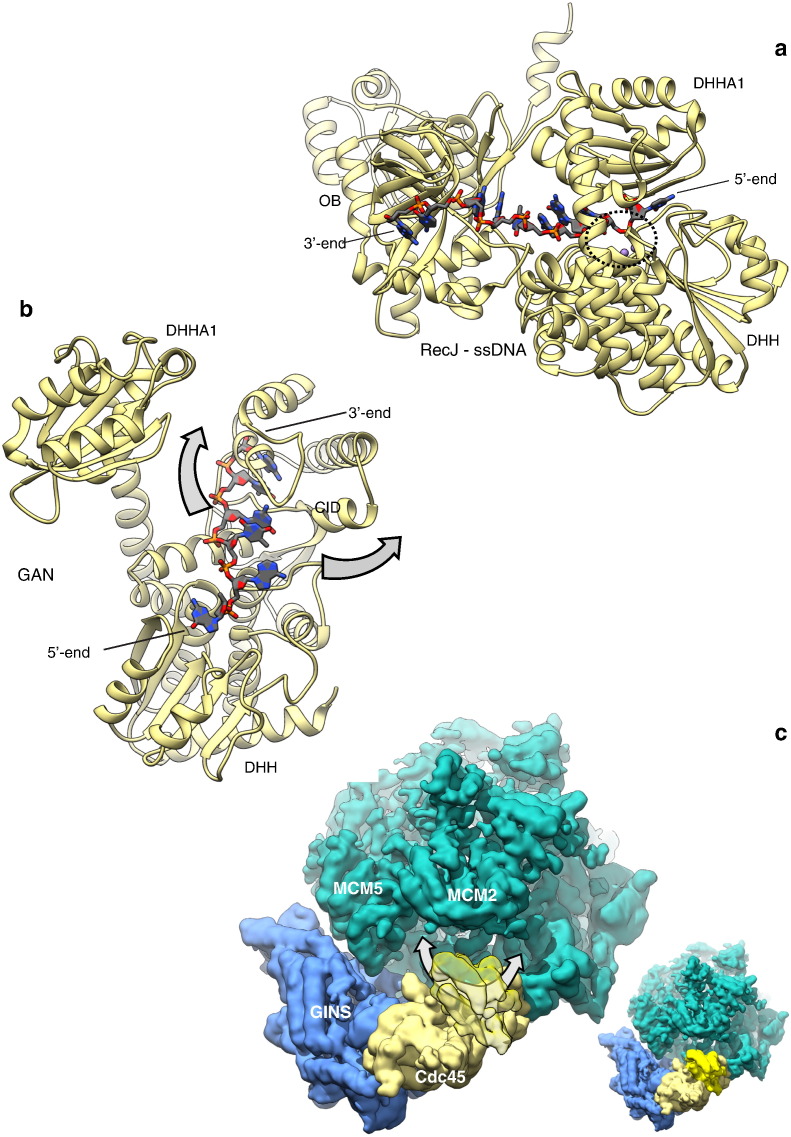
Structure of bacterial RecJ bound to ssDNA and possible insights into the interaction of archaeal GAN with DNA. a Crystal structure of *Deinococcus radiodurans* RecJ bound to ssDNA (PDB ID 5F56). The position of the active site is marked by a dashed oval. b Structure of archaeal GAN (PDB ID 5GHT), with overlaid ssDNA from the superimposed structure of the RecJ-ssDNA complex. The arrows show proposed alternative paths for ssDNA, to avoid a steric clash with GAN's CID. c Cryo-EM map of yeast CMG, coloured according to its components, MCM (green), GINS (light blue) and Cdc45 (pale yellow). The segmented map volume corresponding to the DHHA1 domain of Cdc45 is transparent, to uncover the putative ssDNA site on GANS and Cdc45. The arrows indicate possible trajectories of ssDNA towards the archaeal/eukaryotic MCM ring. The inset in the right-bottom corner shows the same view of CMG, with Cdc45’s DHHA1 highlighted in yellow.

Possible insights into the mechanism of GAN interaction with ssDNA can be gleaned from the recently determined co-crystal structure of *Deinococcus radiodurans* RecJ bound to a oligodeoxynucleotide substrate [14]. The structure shows that the DNA is threaded through the groove between DHH and DHHA1 domains (Fig. 3a); the presence of a phosphate-binding site above the active site help explain RecJ's 5′-to-3′ polarity of resection, while stacking interactions of aromatic side chains with the bases of the DNA contribute to the processivity of ssDNA resection.

Given the remarkable degree of GAN's structural similarity with RecJ and same 5′-to-3′ polarity of digestion, it is reasonable to assume that the RecJ-ssDNA provides a reliable template to predict the geometry of the GAN-ssDNA interaction. One important difference concerns the path of ssDNA as its 3′-end emerges from the back of the enzyme. Whereas in RecJ the 3′-DNA end engages in further interactions with an OB domain, the same trajectory in GAN would lead to a steric clash with its CID (Fig. 3b). Intriguingly, the observation that the position of the DHHA1 is not fixed, due to the considerable flexibility of the long alpha helical stem connecting it to the DHH domain, suggests the possibility that ssDNA might be threaded through an opening between the helical stem and the CID. When considered in the context of an archaeal CMG, this trajectory would take the ssDNA path towards the MCM ring. Alternatively, the path of ssDNA might follow the opposite direction, being threaded over GAN's CID surface instead (Fig. 3b).

Further extrapolation of these considerations to Cdc45 and the eukaryotic CMG would require in the first place a conformational change leading to the physical separation of the DHH and DHH1 domains, to allow Cdc45 to engage with ssDNA. No evidence for such rearrangement is available at the moment, although it should be noted that Cdc45 is poised to interact with other components of the replisome, beyond MCM and GINS, that might conceivably drive such change, under specific yet-unknown circumstances. Of note, Cdc45’s tertiary structure contains conserved elements of secondary structure, such as the long helical insertion in its DHH domain (Fig. 1c), that are not explained by the structural context provided by the CMG. Should the DNA-binding groove of Cdc45 become available and interact with DNA as observed for RecJ, a DNA strand threading through Cdc45's inter-domain groove would be on a path towards the MCM assembly, as proposed for GAN. Notably, such a path might take the ssDNA towards the MCM2 and 5 interface, a dynamic boundary that allows topological transactions of fork DNA strands in and out of the MCM ring.

## 5 Summary

In summary, these recent findings define at atomic level the Cdc45/RecJ fold, explain how Cdc45 interacts within the eukaryotic CMG and highlight some unexpected features of the Cdc45 structure that are likely to be relevant for replisome function. They further show that the GAN nuclease must be considered a *bona fide* component of the archaeal CMG, and consequently a role for its nuclease activity is likely to exists in DNA replication. The recent reconstitution and biochemical characterization of an archaeal CMG [19] bodes well for the discovery of what the function might be. The structure of RecJ in the act of 5′-DNA resection provides a possible structural template to begin to unravel the structural basis for the interaction of the Cdc45/RecJ/GAN family of proteins with DNA. Finally, whether all, part or none of the recent experimental observations made for GAN and RecJ are applicable to Cdc45, in the manner speculated here, will be a fascinating subject of future investigations.
